# More than Half of High School Students Report Disordered Eating: A Cross Sectional Study among Norwegian Boys and Girls

**DOI:** 10.1371/journal.pone.0122681

**Published:** 2015-03-31

**Authors:** Monica Klungland Torstveit, Kjersti Aagedal-Mortensen, Tonje Holte Stea

**Affiliations:** University of Agder, Faculty of Health and Sport Sciences, Kristiansand, Norway; University of Bremen, GERMANY

## Abstract

Disordered eating and eating disorders are of great concern due to their associations with physical and mental health risks. Even if adolescence has been identified as the most vulnerable time for developing disordered eating, few studies have used a broad spectrum of criteria to investigate the prevalence of disordered eating among high school students of both genders, in different programs of study, nor assessed correlates of disordered eating among this important target group. The purposes of this study were therefore to investigate the prevalence and correlates of disordered eating among both male and female high school students in sport-, general and vocational programs. A comprehensive questionnaire was completed by 2,451 students (98.7%), aged 15–17 years. The total prevalence of disordered eating was 54.9%, with 64.3% among girls and 45.0% among boys (p<0.001). The highest prevalence of disordered eating was found among vocational students (60.7%), followed by students in general programs (49.8%) and sport students (38.3%) (p<0.001). Female gender, school program (vocational and general), overweight/obesity and weight regulation were positively associated with disordered eating. The high prevalence indicates the importance of tailored prevention efforts directed at high school students, particularly in vocational programs. Furthermore, a smaller girls–boys ratio than expected indicates that the efforts to identify and manage disordered eating among high school students should include both genders.

## Introduction

Disordered eating (DE) behaviors may include a preoccupation with body weight and shape, food restriction, dieting, binge eating, vomiting, and abuse of diuretics, laxatives and diet pills, which may serve on a continuum preceding clinical eating disorders (EDs) [1, 2]. As a result of the biological changes, peer pressure, societal drive for thinness and a body image preoccupation that occurs during puberty, adolescence is pointed out as the most vulnerable time for developing DE [3, 4], and it is suggested that adolescents account for 40% of new cases of EDs [5].

Previous studies indicate a wide gender gap in terms of EDs, with a high female:male ratio in general (9:1 or 10:1) (e.g. [6, 7]). However, this ratio seems to be smaller among the high-risk group of adolescents, varying from 3:1 to 10:1 [8–10]. When it comes to DE behaviors which typically precede clinical EDs, girls seem to outnumber boys to an even lesser extent [11, 12], but few studies have addressed this among adolescents.

The prevalence rate of DE among high school students in previous studies has varied considerably, which is probably due to the use of different methodologies and different definitions of DE. Most of these studies have used only one or a few screening instruments or criteria to define DE [12–18], thereby increasing the risk of false negative subjects. Furthermore, only a few studies have included adolescents of both sexes (e.g. [15, 17–20]).

Over the last 30 years, an increased attention directed towards a high prevalence of DE and EDs among athletes has evolved [21–25], though it is not clear whether being an athlete is an independent risk factor regarding the development of DE [26]. In two recent studies, a lower prevalence of DE among adolescent athletes compared to non-athletes has been obtained [27, 28]. A reduced risk for DE has also been indicated in a meta-analysis including non-elite athletes, especially those in high school, compared to non-athletes [21]. However, these latter mentioned results are only based on a few studies, mostly focusing on female high school athletes, and with small control groups. Moreover, prevalence studies of DE among other groups of high school students attending different study programs are lacking.

A recently published study, including a nationally representative sample of more than 14,000 US males and females, concluded that the transition period from adolescence to young adulthood is critical for assessing and preventing weight- and eating related problems [29]. A Norwegian population based longitudinal study suggests that initial DE is a stronger predictor in late rather than in mid-adolescence [30]. Furthermore, Neumark-Sztainer et al. [31] have demonstrated that the high prevalence of DE behaviors among adolescents continues through adulthood, and that individuals who practice DE behaviors during adolescence have an increased risk for the continued use of these harmful behaviors 10 years later. In order to determine where to direct the preventive work, it is therefore also essential to examine the correlates of DE among adolescents. Dieting has been shown to increase significantly from 1992 to 2010 among both adolescent boys and girls in Norway [32]. Dieting has also been implicated as a potential risk factor in the development of DE and EDs [10, 33,34]. One example is a study by Neumark-Sztainer et al. [34], showing that adolescent dieters at the 5-year follow-up were at a significantly higher risk of DE, such as vomiting or the use of diet pills or laxatives, than non-dieters. While the body ideal for women is slender, the current masculine ideal seems to be both lean and muscular, thus possibly leading to potentially harmful behaviors such as excessive exercise to gain muscle and weight regulation to both lose and gain weight [35, 36]. The association between a drive for muscularity and/or weight gain and DE among adolescents has been understudied, but a recent French study concluded that the pursuit of muscularity was related to DE among French adolescent boys, and that both a drive for thinness and a drive for muscularity need to be considered when examining body concerns among boys and girls [37]. Furthermore, a Norwegian study concludes that muscularity measures should be included in the screening for EDs in adolescent boys [38].

Some studies have shown a higher prevalence and/or a higher risk of DE among overweight or obese adolescents compared to normal weight adolescents [39–42]. These studies, however, have only used one screening instrument to define DE, thereby increasing the risk for false negative subjects.

DE and EDs are of great concern due to their associations with physical and mental health risks [43–45]. In a review, Chamay-Weber et al. [45] reported that adolescents who did not meet the diagnostic criteria for clinical EDs, but who were engaged in DE behaviors, were more likely than adolescents without DE to report mood disorders such as depression, anxiety, and substance use and misuse. An increased risk for suicidal behavior has also been reported among adolescents engaging in DE behavior such as self-induced vomiting and laxative use for weight control purposes [46]. These findings indicate that potentially serious health risks exist among adolescents with DE behavior, even if they do not meet the diagnosis of a clinical ED, thereby underscoring the need for the screening and prevention of these harmful behaviors and disorders.

Therefore, the main purposes of the present study were to investigate the prevalence of DE, using a broad spectrum of criteria among high school students, and to conduct comparisons between students in sport-, vocational- and general programs, as well as between genders. Finally, it was of interest to assess correlates of DE, such as weight categories and weight regulation, among the participating sample. We hypothesize that the prevalence of DE in this adolescent group in general is high, higher among girls than boys, and lower among students in sport programs than students in vocational- and general programs. We also hypothesize that being overweight/obese and having a history of weight regulation increase the odds for having DE.

## Materials and Methods

This study is part of a larger school-based cluster randomized intervention study, “Active and Healthy Youth”, promoting a healthy diet and activity patterns that aim to prevent DE among Norwegian adolescents.

### Participants

**Fig 1:**
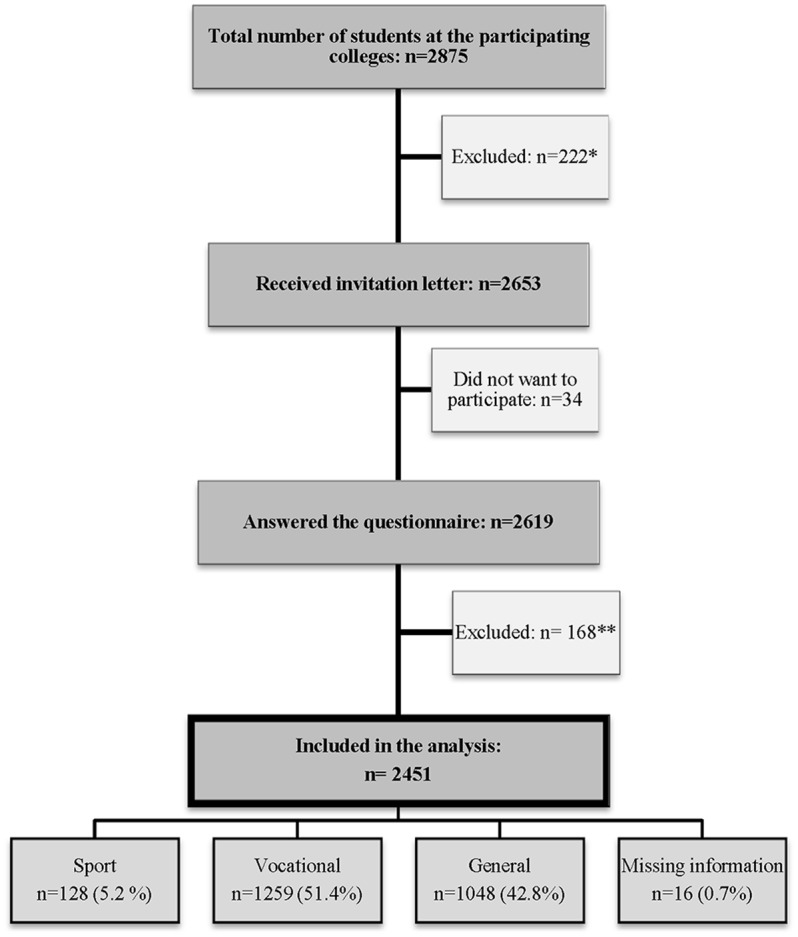
Flowchart showing inclusion and exclusion of participants in the study and classification of the participating sample. *Had dropped out from school, school classes that participated on excursions/project work or absence due to illness, travel or other unknown reasons. **Age >17 years.

The target group was the total population of 1^st^ year students at all high schools in two counties in Southern Norway, aged 15–17 years, with a total of 17 of 23 schools agreeing to participate in the study (73.9%). The reasons why six schools chose not to participate were a lack of time (five schools) or a present participation in another research study (one school). Hence, the participating sample consisted of 17 schools, while the number of 1^st^ grade students available at data collection was 2653. Of these, 2619 students chose to participate (98.7%). When analyzing data in the present study, 168 students were excluded because they were over 17 years old. The participants in this study therefore consisted of 2451 students aged 15–17 years (Fig 1).

In Norway, high school students choose a study program according to their area of interest. Thus, the students were classified into one of three categories according to their program of study: sport, vocational (e.g. construction, industry, health care) or general (Fig 1).

### Procedure

The data collection was carried out in classrooms or auditoriums, and at least one member of the project team was continuously present to inform about the project and to answer possible questions. Written consent was obtained from the adolescents prior to participation in the study. Confidentiality and privacy were ensured by having no names or other identification on the questionnaires, and by placing students so far apart while they answered the questionnaire that they could not talk to each other or see others’ responses. Teachers and project staff were present to assure that the students followed these procedures.

The study has been approved by the Norwegian Regional Committee for Medical Ethics South-East (ref: 1348b). This is an independent committee, appointed by the Norwegian Ministry of Education. In Norway, written parental consent is not required by the Committee for Medical Ethics for adolescents aged 15 years and older. As all participants in the present study were 15–17 years old, no parental consent was needed. Written, informed consent was, however, obtained from all the participants. They were encouraged to inform their parents about their participation and show them the information letter that described every detail of the study. Furthermore, the data collection was conducted during school time and principals, teachers and school nurses were given written information about the study. In addition, the study was approved by all principals and school boards.

### Measures

#### Questionnaire

The participants were asked to complete a questionnaire, including both standardized tests such as the Eating Disorder Inventory-2 (EDI-2) [47] and the Drive for Muscularity Scale (DMS) [48], as well as self-developed questions measuring weight regulation and symptoms related to DE.

The EDI-2 [47] is a self-report screening instrument designed to assess attitudes, feelings and behaviors associated with EDs [49], and is one of the most widely used measures of ED symptoms [50]. The EDI has been found valid in clinical and non-clinical female samples [49] and suitable for use as a screening instrument for EDs in a non-clinical setting [51]. It is therefore concluded that the EDI-2 discriminates well between ED patients and both psychiatric outpatients and controls in general [49]. The EDI-2 consists of 11 subscales, of which the questionnaire used in the present study included the three eating-disorder-specific subscales (in contrast to the other general psychological scales relevant, but not specific, to eating disorders): Drive for Thinness (EDI-DT), Body Dissatisfaction (EDI-BD) and Bulimia (EDI-B). These three are the subscales of the EDI-2 that have been found to be most strongly correlated to EDs [52], and the same subscales were also used in a recent comparative study investigating DE behaviors among Cypriot adolescent boys and girls [53]. Additionally, several studies have used the subscales EDI-BD and EDI-DT to also identify DE or EDs among both adolescent and young adult athletes (e.g. [22, 24, 27, 54–56]). The reliability of the EDI-2 subscales used in the present study is found to be relatively high, with test-retest correlations ranging between. 86 and. 89 among a sample of ED inpatients, and between. 91 and. 94 among patients suffering from diseases other than EDs [57]. A strong reliability of EDI-DT and EDI-BD has also been identified among adolescents, with an internal consistency between. 89 and. 92 [58].

Since the EDI subscales primarily focus on body dissatisfaction related to thinness, and since the drive for thinness seems to be relatively rare in boys and men [59], the DMS was included in our questionnaire. The DMS is a 15-item self-report questionnaire divided into the subscales of Body Image (DMS-BI) and Behavior (DMS-B), which assesses attitudes and behaviors related to the desire for a muscular body. The DMS has been proven to be valid for assessing the drive for muscularity in both boys and girls [48, 60, 61], and has revealed a satisfactory reliability with alphas for boys and girls of. 84 and. 78, respectively [48].

The self-developed questions about dieting history and ED history were included based on use in previous studies on adolescent or young adult female and male athletes and non-athletes (e.g. [23, 24, 27]). Body mass index (BMI) (kg/m^2^) was calculated based on self-reported height and weight, and used to classify underweight among the participants, using the age-adjusted values suggested by Cole et al. [62]. In order to categorize the overweight and obese students, sex- and age-specific International Obesity Task Force (IOTF) cut-off points for defining overweight and obesity in children and adolescents aged 2–18 were used [63].

#### Symptoms of disordered eating

Criteria for being classified with DE in this study are based on previous studies (specified in Table 1), and are as follows: (a) high scores on the EDI subscales, i.e. EDI-DT (girls ≥15, boys ≥10), EDI-BD (girls ≥14, boys ≥ 10), and EDI-B (girls ≥7, boys ≥5), (b) age-adjusted underweight BMI values (girls <17.9 kg/m^2^, boys <17.5 kg/m^2^), (c) ‘‘self-reported EDs,” i.e. a subjective experience of past or current EDs, (d) trying to lose weight three times or more in the past, (e) scores on the DMS subscales exceeding the 90^th^ percentile of the sample, and (f) the use of pathogenic weight control methods (PWCM) (diet pills, hunger-repressive pills, laxatives, diuretics, or vomiting).

**Table 1:** Criteria for being classified with disordered eating in the present study and references to previous studies these criteria are based on.

	Boys	Girls	References
EDI-DT	≥ 10	≥ 15	(e.g. [24,27,55,56])
EDI-BD	≥10	≥ 14	(e.g. [24,27,55,56])
EDI-B	≥ 5	≥ 7	[47,64]
BMI kg/m2	<17.5	<17.9	[27,62]
DMS-BI	≥ 37	≥ 26	[90^th^ percentile of the total sample]
DMS-B	≥ 28	≥ 19	[90^th^ percentile of the total sample]
Tried to lose weight ≥ 3 times before			(e.g. [24,27])
Self-reported ED (a subjective experience of past or current ED. Answers “yes” or “I don’t know”)			(e.g. [24,27,55,56])
Use of PWCM (diet pills, hunger-repressive pills, laxatives, diuretics or vomiting)			(e.g. [24,27,55,56])

### Statistical analysis

All statistical analyses were performed using SPSS version 19.0 (SPSS Inc., Chicago, IL). The results are presented as the mean value and standard deviation (SD) for continuous data, and absolute numbers and percentages for categorical data. BMI was normally distributed in our group of adolescents. An independent samples t-test was carried out to compare BMI between genders, whereas one-way ANOVA and Bonferroni post-hoc tests were conducted to compare BMI values between students attending different study programs. A chi square test (χ 2) was used to compare DE between genders as well as between students attending different study programs. Multiple logistic regressions were used to explore the relationship between DE and program (sport, vocational and general), gender (boys, girls), weight-categories (normal weight/underweight, overweight/obese), and weight regulation (tried to lose/gain weight). Odds ratios are presented with 95% confidence intervals (CI). A two-tailed p-value of <0.05 was considered statistically significant.

## Results

### Subjects characteristics

The mean age of the participating sample was 16.0 years (SD: 0.4 years), while the mean BMI was 21.7 (4.2) kg/m^2^ for the girls and 22.2 (3.7) kg/m^2^ (p = 0.004) for the boys (Table 2).

**Table 2:** Body Mass Index (BMI) values and prevalence of disordered eating (DE) and the different symptoms among girls and boys presented by program in high school.

BMI and symptoms of DE	GIRLS				BOYS			
	All girls	S	V	G	All boys	S	V	G
Number (n)	n = 1254	n = 74	n = 581	n = 599	n = 1181^†^	n = 54	n = 678	n = 449
**Mean BMI (kg/m** ^**2**^ **) (SD)**	21.7 (4.2)*	22.5 (6.6)	22.0 (4.5)	21.4 (3.6)	22.2 (3.7)	21.5 (1.8)	22.5 (4.1)**	21.9 (3.1)
**Total DE symptoms** ***	64.3	50.0	72.2	58.3	45.0	22.2	50.9	38.5
***EDI-DT ≥ cutoff***	13.2	8.1	15.3	11.7	3.4	-	3.8	3.3
***EDI-BD ≥ cutoff***	31.1	17.6	36.5	27.5	15.0	-	19.5	10.5
***EDI-B ≥ cutoff***	5.2	1.4	8.4	2.5	5.8	-	7.4	4.2
***BMI ≤cutoff***	7.1	1.4	8.7	6.2	3.4	1.9	4.0	2.9
***Dieting*** ^***1***^	38.8	28.4	44.3	34.6	11.1	3.7	12.7	9.4
***PWCM*** ^***2***^ ***(total)***	15.6	4.1	22.6	9.8	5.3	-	6.8	3.6
***Diuretics***	5.8	-	9.1	3.0	3.4	-	4.7	1.8
***Laxatives***	5.0	-	8.2	2.2	3.2	-	4.1	2.0
***Vomiting***	9.8	4.1	13.9	6.2	3.2	-	4.3	1.8
***Diet pills***	7.8	-	12.2	4.2	3.5	-	4.6	2.2
***Self-reported ED*** ^***3***^	28.5	21.6	34.8	22.9	14.0	1.9	16.7	11.4
***DMS BI ≥ cutoff***	8.4	6.8	10.5	6.7	9.8	5.6	9.7	10.5
***DMS B ≥ cutoff***	9.3	9.5	10.3	8.0	8.7	14.8	8.7	7.8

Mean values for BMI are presented with standard deviation (SD). Prevalence data are given in percentage (%).

Programs in high school: S: Sport, V: Vocational, G: General.

^†^n = 16 missing information regarding school program.

^1^Tried to lose weight ≥3 times.

^2^PWCM: Pathogenic Weight Control methods.

^3^ED: Eating disorder.

*p = 0.004 compared with the boys.

**p = 0.044 compared with the male students in general programs.

***p<0.001 comparing the different study programs among girls, as well as among boys.

### Prevalence of disordered eating

#### Total sample

The prevalence rate of DE was 54.9% in the total sample, with a significantly higher number of girls (64.3%) compared to boys (45.0%) reporting DE (p<0.001) (Table 2).

#### Study programs

The highest prevalence of DE was found among vocational students (60.7%), followed by students in general programs (49.8%), and sport students (38.3%) (p<0.001). Comparing the prevalence of total DE symptoms between students in the three different study programs there was an overall significant difference among the girls (p<0.001) and among the boys (p<0.001) (Table 2).

#### Individual symptoms of disordered eating

A total of 21.5% of the students (Girls (G): 28.5%; Boys (B): 14.0%) reported a current or previous ED (“yes” or “I don’t know”), 25.3% (G: 38.8%; B: 11.1%) had attempted to lose weight three or more times and 10.6% of the students (G: 15.6%; B: 5.3%) reported use of PWCM. Table 2 gives a further description of the individual symptoms of DE among female and male students in the different study programs.

### Correlates of disordered eating

Compared to students in the sport programs, the results show a higher odds for DE among the students in vocational programs (OR: 2.8, 95% CI: 1.8–4.4) (p<0.001) and general programs (OR: 1.6, 95% CI: 1.0–2.5) (p = 0.032), and an increased odds for DE was shown among girls compared with boys (OR: 2.5, 95% CI: 2.1–3.0) (p<0.001). A higher odds for DE was found among the overweight/obese compared with the normal weight/underweight students (OR: 2.3, 95% CI: 1.7–3.1) (p<0.001) (Table 3). Stratifying the sample into girls and boys, adjusting the analysis for BMI, and including study programs and dieting (“have you ever tried to lose weight?”) as covariates, further analyses showed that dieting was significantly associated with DE in girls (OR: 6.7, 95% CI: 4.9–9.0) and boys (OR: 4.1, CI: 3.0–5.7) (p<0.001) (Table 4).

**Table 3:** Association between disordered eating as the dependent variable and high-school program, gender and weight category as the independent variables.

	B	Odds ratio (95% CI)	p-value
Vocational^1^	1.055	2.8 (1.8–4.4)	<0.001
General^1^	0.471	1.6 (1.0–2.5)	0.032
Girls^2^	0.916	2.5 (2.1–3.0)	<0.001
Overweight/obese^3^	0.045	2.3 (1.7–3.1)	<0.001

^1^Sport was used as a reference.

^2^Boys was used as a reference.

^3^Normal weight/underweight was used as a reference.

**Table 4:** Association between disordered eating as the dependent variable and high school program and weight regulation among girls and boys as the independent variables.

	B	AOR^a^ (95% CI)	p-value		B	AOR^a^ (95% CI)	p-value
**Girls**				**Girls**			
Vocational^1^	0.695	2.0 (1.1–3.6)	0.019	Vocational^1^	0.985	2.7 (1.6–4.5)	<0.001
General^1^	0.000	1.0 (0.6–1.8)	0.996	General^1^	0.276	1.3 (0.8–2.2)	0.298
Dieting^2^	1.895	6.7 (4.9–9.0)	<0.001	Weight increase^3^	0.417	1.5 (1.1–2.2)	0.030
**Boys**				**Boys**			
Vocational^1^	1.065	2.9 (1.5–5.7)	0.002	Vocational^1^	1.277	3.6 (1.8–7.0)	<0.001
General^1^	0.572	1.8 (0.9–3.5)	0.105	General^1^	0.809	2.3 (1.1–4.5)	0.020
Dieting^2^	1.414	4.1 (3.0–5.7)	<0.001	Weight increase^3^	0.745	2.1 (1.6–2.8)	<0.001

^a^Adjusted for Body Mass Index (BMI)

^1^Sport was used as a reference.

^2^Have you ever tried to lose weight?,

^3^Have you ever tried to gain weight?

By replacing the dieting variable with a weight increase variable (“have you ever tried to gain weight?”), the results showed that weight gain was significantly associated with DE in girls (OR: 1.5, 95% CI: 1.1–2.2) (p = 0.030) and boys (OR: 2.1, 95% CI: 1.6–2.8) (p<0.001) (Table 4).

## Discussion

### Prevalence of disordered eating

We found that 55% of the high school students reported DE, which is somewhat higher compared to results from previous studies (e.g. [13, 14, 18, 28]). This is probably due to the different criteria for DE used in the different studies. In order to avoid false negative subjects, we included several criteria such as underweight, dieting history, self-reported ED, use of PWCM and the DMS, in addition to subscales of the EDI-2. A very similar range of criteria was used in a recent study investigating DE among high school elite athletes and non-athletes in Norway [27]. They also reported a high prevalence of DE among the high school athletes (29%) and an even higher percentage among the non-athletic high school students (51%). Gonsalves et al. [65] noticed that more than 70% of students in both middle and high school reported using methods to control or maintain their weight, including unhealthy methods such as fasting, vomiting, laxative use or taking diet pills without a doctor’s permission. Based on these studies, one can question whether being obsessed with body weight and shape, appearance, dieting or muscle weight gain, exercise and/or food/eating may be a more normal than seldom phenomenon in today’s society. Nevertheless, in the authors’ opinion, the inclusion of several criteria for being identified with DE is important, particularly among adolescents, to help identify every girl and boy with DE, thus giving health-care providers the opportunity to provide appropriate guidance or treatment to all adolescents suffering from DE, and possibly prevent the development of more serious EDs.

The second aim of the present study was to conduct comparisons of the prevalence of DE between students in sport-, vocational- and general programs, and between genders. We found a significantly lower prevalence of DE among sport students (38.3%) compared to students in vocational (60.7%), and general (49.8%) programs. Additionally, when investigating individual risk criteria, such as use of PWCM or a high score on the EDI subscales, we found that the sport students reported less symptoms compared to students in the other study programs. These results are consistent with a tendency observed in two comparable studies [27, 28], in which a lower prevalence of DE among high school athletes at different levels compared to non-athletes was shown. Furthermore, a meta-analysis based on 34 studies [21] has also described a tendency towards a reduced risk of DE among high school non-elite athletes compared to non-athletes. On the other hand, a more recent review of 22 studies revealed no significant differences in the prevalence of DE between female athletes and controls aged 12–35 years in 12 of them [25], indicating a similar risk of developing DE between the two groups. Supportive of these results, a study by Monthuy-Blanc et al. [66] revealed an equivalent frequency of DE among adolescent female athletes and non-athletes.

In our study, a surprisingly high number of vocational students reported DE (6 out of 10). A total of 10%, 28%, and 8% in this group scored above the cutoff on the EDI-DT, the EDI-BD and the EDI-B subscale, respectively. Almost one in three (29%) had attempted to lose weight three times or more, and a total of 15% had used PWCM to lose weight. In addition, 26% of the vocational students reported an ED. These findings are alarming considering the young age of these subjects, which will be further discussed under the Correlates of DE. To the best of our knowledge, no other studies have investigated the prevalence of DE among vocational students.

In terms of gender differences, we found a higher prevalence of DE among girls (64%) than among boys (45%). These results are consistent with other studies showing that girls seem to be at higher risk than boys for developing DE (e.g. [13, 18, 28]). Despite this gender difference, the rate itself suggests a relatively small gap between girls and boys in our sample (female-male-ratio: 1.4:1) compared to the previously mentioned female–male-ratio of EDs (3:1 to 10:1) among adolescents [8–10]. Our finding of a small gender gap is supported by some previous studies (e.g [13, 27, 28, 65]), but these studies still have a slightly higher ratio than what was found in the present study. The fact that the female-male ratio of DE found in the present study is smaller than the female-male ratios presented in previously published studies may be partly explained by the inclusion of DMS as one of the measurement instruments, hence possibly resulting in more true positive subjects, especially among boys. Hautala et al. [12] further suggested that gender differences are relatively smaller concerning milder forms of EDs compared to the serious EDs, as well as being smaller among adolescents compared to adults. The relatively small gender gap may also simply indicate an increased prevalence of DE behavior among boys compared with girls during the last couple of years.

### Correlates of disordered eating

When examining the relationship between DE and the independent variables study-program and dieting (“have you ever tried to lose weight”) in the present study, dieting proved to be the strongest predictor of DE. We found a 6.0 and a 3.7 times higher odds for DE among girls and boys with a dieting history, respectively, compared to those without such a history. These results support findings from previous studies, showing a strong relationship between dieting and DE (e.g. [28, 67]). It should be noted, that in our study, one of the nine criteria for defining DE was trying to lose weight three times or more in the past. This variable may correlate with the independent variable “have you ever tried to lose weight”. Additional analysis revealed, however, that of those students who reported that they had tried to lose weight, as many as 40.8% of the girls and 59.1% of the boys had tried to lose weight once or twice and were therefore not included in the dieting criteria for DE. Our results indicate an increased odd for DE among both girls and boys who had tried to reduce or increase weight only once previously. This suggests that we must not look only for the most extreme dieters in order to identify adolescents at risk for DE. Taking into account the recent findings among Norwegian adolescents, of how secular trends in eating problems are related to changes in putative risk factors, including dieting [32], it may be of value to focus on preventing unnecessary dieting in this young group.

By replacing the variable “dieting” with the variable “weight increase” (“have you ever tried to gain weight”), we found that girls and boys reporting attempts of gaining weight had 1.5 and 2.1 times higher odds of DE, respectively, compared to those who had not attempted to gain weight. Assuming that the desire to gain weight is a desire for increased muscle mass, these results are in agreement with a recent study among French adolescent boys [37], suggesting that a relationship between a drive for muscularity and DE exists. Furthermore, higher EDI subscale scores and lower DMS score have been found among adolescent girls compared to boys, whereas correlation between EDI subscale scores and DMS have been detected among adolescent boys only [38]. These findings highlight the importance of including drive for muscularity as a criterion in studies investigating prevalence of DE. However, to further investigate this relationship, it will be necessary to perform longitudinal studies in the future.

An increased odds for DE was shown among girls compared with boys in our study. This result is in line with the results presented by Martinsen et al. [27], which showed a five times higher prevalence of DE among female high-school elite athletes and nearly eight times higher prevalence of DE among female controls, compared to the male counterparts. Furthermore, the students attending vocational- and general programs in our study showed 2.8 and 1.6 times higher odds for having DE, respectively, compared to those attending the sport program. These results support previous findings of a lower frequency of DE among high school athletes at different levels compared to non-athletes [21, 27, 28] and may indicate that being a sport student at a high school may be somewhat protective against DE.

We found that the overweight/obese students had 2.3 times higher odds for DE compared with the normal weight/underweight students. This is in accordance with Musaiger et al. who found a two to three times higher risk for DE among overweight/obese compared to non-overweight/obese boys and girls in seven Arab countries [40]. Corresponding values have also been found among Mexican adolescents [41] and among adolescents in high schools in Tehran [39]. Neumark-Sztainer et al. [68] found that American overweight adolescents report being teased about their weight and being bothered by the teasing, and further that weight-teasing is associated with DE behaviors that may place overweight youth at increased risk for weight. The importance of approaching both DE and overweight/obesity among adolescents therefore seems to be of high importance.

Our findings further indicate that attending vocational programs in high school may be a possible risk factor for developing DE. Yet, it should be taken into consideration that due to the cross-sectional study design, it is not possible to determine whether the onset of DE symptoms occurred before or after the students started high school. Considering the fact that indoctrinations of the thin body ideal seem to occur as early as 6 years of age [69], and that concerns about body fat and dieting have been revealed in a high number of 8–13 year old girls and boys [70], it is possible that the students identified with DE symptoms in the present study also had these symptoms before starting high school. That being the case, one may ask why so many of those adolescents select vocational programs. Longitudinal studies initiated before students enter high school are needed in order to answer these questions.

### Criteria for disordered eating

The subscales EDI-DT and EDI-BD, as well as BMI, dieting, use of PWCM and self-reported EDs, have been used as criteria for DE in several previous studies (e.g. [22–24, 27, 55, 56]). In contrast, limited research using cutoff scores to the subscale EDI-B exists, especially among girls, and the cutoff score used for boys in the present study was based on results from a previously published study [64]. Moreover, because the cutoff scores for EDI-DT and EDI-BD have been higher for girls than for boys in comparable studies (e.g. [24, 27]), the cutoff score for the EDI-B subscale was also set higher for girls than for boys in the present study, using appendix A7 in the EDI-2 manual [47] as a norm. Dieting is one of the most important risk factors for developing EDs [10, 32, 71], and weight loss should in some way be included as one of the indicators of DE. However, the definition of subjective dieting among adolescents may vary. Some could define their attempt at eating healthier food as dieting, whereas others may not use the definition of “dieting” before they reach a level almost equivalent to fasting. As a result, studies using different definitions may be biased with false positive and/or false negative results. In the present study, in order to reduce the risk of false positives, only the participants who reported having attempted to lose weight ≥ 3 times were defined as having symptoms of DE. Because the use of PWCM was also included as an indicator of DE, it was assumed that the most extreme dieters would be identified. Furthermore, as mentioned above, the question, “Have you ever tried to lose weight?” was examined as a possible predictor for DE, which will also include those students trying to lose weight for the first time.

### Strengths and limitations

A major strength of the present study was the very high response rate (98.7%). In addition, no geographical or socioeconomic differences were found comparing the participating schools to the non-participating schools. Thus, the external validity of our findings was enhanced by the representativeness of the sampling strategy and the size and diversity of the sample.

However, there was an uneven distribution of students between the three programs, as the number of sport students was significantly smaller (n = 128) than the number of students in vocational (n = 1259) and general (n = 1048) programs. Nonetheless, these numbers reflect reality, since no other participating school offered sport programs.

The use of self-reported data, including BMI, as a basis for investigating the prevalence of DE is a limitation of the present study and eliminates the ability to diagnose clinical EDs. A recent work by Martinsen and co-workers indicates that the prevalence of DE among adolescent elite athletes is lower compared to non-athletes [27], although the prevalence of clinical EDs is higher among adolescent elite athletes compared to non-athletes [72]. Still, whether the same pattern also exists among the sample in our study is unknown, and needs further investigation using clinical interviews.

### Future implications

The high prevalence rate of DE shown in the present study should be taken seriously, as it gives an indication of a society in which being obsessed about body weight and shape, dieting, muscle weight gain and appearance may represent the norm among adolescent girls and boys. However, our results indicate that being a high school student in the sport program, compared to the vocational or the general program, may be somewhat protective regarding the development of DE. For this reason, encouragement for sports participation and an increased physical activity level should be performed as early as from childhood. Surprisingly, we found an alarmingly high prevalence rate of DE among students participating in vocational programs. To the best of the authors’ knowledge, this group has not previously been identified as being at increased risk for developing DE or EDs. Further research is needed to identify the important determinants for developing DE, as well as effective interventions tailored for this important target group. The fact that the girls showed a higher prevalence of DE compared to the boys in our study is an indication that gender differences still exist. Nevertheless, the girls-boys ratio was lower than anticipated. This can be explained by high prevalence rates of DE among boys, particularly those attending vocational programs, which indicates that the identification and further management of DE among high school students must include both genders.

Lastly, it is suggested that the use of DE behaviors is likely to set the stage for the continued use of these behaviors later on [31] and that DE during early adolescence predicts later DE, especially in late adolescence [30] A recent study based on a nationally representative sample in the US suggests that adolescents continue to engage in dieting and extreme weight loss behaviors into young adulthood, and that these behaviors become more widespread over time [29]. To reverse this trend, longitudinal studies should be performed to determine the onset of DE behaviors, and thus enable the initiation of prevention work before the onset of these harmful behavioral patterns.

## Conclusions

The results from the present study revealed an overall high prevalence rate (55%) of DE among Norwegian high school students aged 15–17 years. Among the three investigated groups of high school students, the vocational students displayed the highest prevalence of DE, and the sport students the lowest. Higher prevalence rates of DE were identified among girls compared to boys in all study programs, though the girls–boys ratio was relatively low. The variables of gender, dieting and participation in vocational programs were most strongly associated with DE.

## Supporting Information

S1 File Questionnaire used in the main study. The questions are written in Norwegian.
(PDF)
