# Supplementary material for: More than Half of High School Students Report Disordered Eating: A Cross Sectional Study among Norwegian Boys and Girls
Source: PLoS One. 2015 Mar 31;10(3):e0122681. doi: 10.1371/journal.pone.0122681 (PMC4380422; doi:10.1371/journal.pone.0122681)
Supplement: S1 File — The questions are written in Norwegian. (PDF) [file pone.0122681.s001.pdf]

ID

Prosjekt

"Aktiv Ungdom med Overskudd"

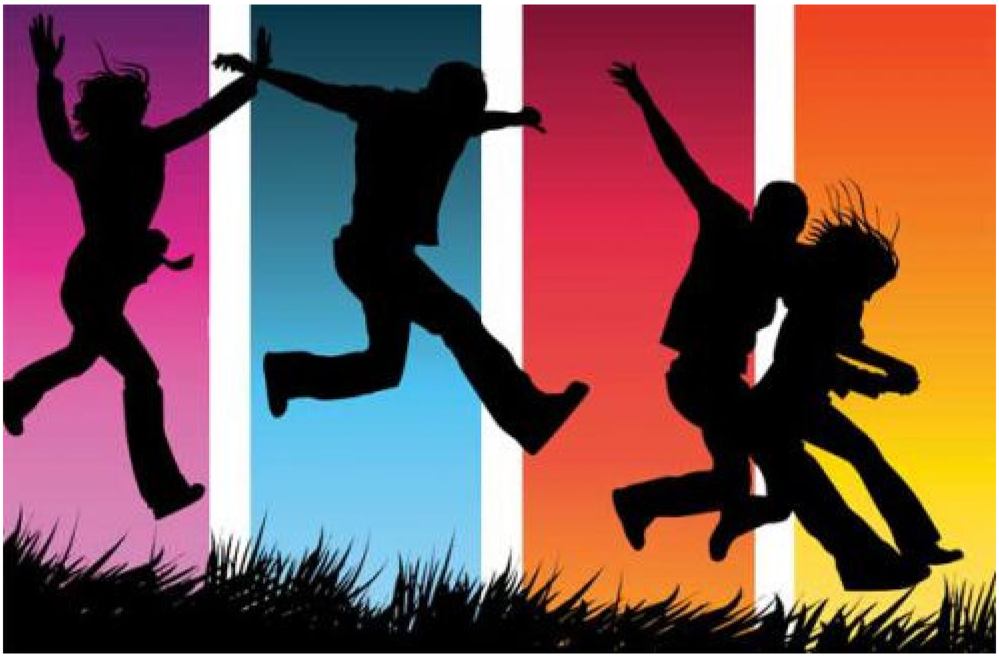

http://s3.hubimg.com/u/2371578_f496.jpg

Spørreskjema

Kjære elev! Les dette først!

Vi synes det er flott at du vil delta i prosjektet ”Aktiv Ungdom med Overskudd”!

På de neste sidene følger noen spørsmål om fysisk aktivitet, kosthold, kropp og helse. Du skal sette kryss ved det svaret som passer best for deg. Det er viktig at du leser spørsmålet og svarene nøye før du setter kryss.

Vær oppmerksom på at spørreskjemaet har spørsmål på begge sider av arket.

Dersom du ønsker å forandre et svar etter at du har satt kryss, – sett da strek over krysset, og sett et nytt kryss på det svaret som passer best.

Husk dette før du setter i gang: ***Vær ærlig! Det er ingen svar som er mer riktige enn andre, og ingen får vite hva du har svart.***

Lykke til med utfyllingen av spørreskjemaet, det vil ta deg ca. 20 minutter!

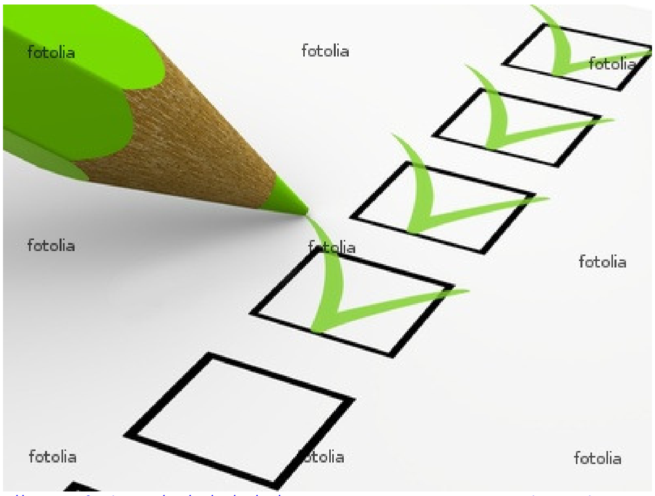

http://static-p3.fotolia.com/jpg/00/07/82/96/400_F_7829651_CywjcsBRzx2e6GnNObyCAzaCkP3L40R7.jpg

1. Kjønn: ☐ Jente/kvinne ☐ Gutt/mann

2. Hvor gammel er du? ☐☐ År

3. Hvilken utdannelse har foreldrene dine? (Sett ett kryss for høyeste utdannelse for far og ett kryss for høyeste utdannelse for mor).

**Far**	**Mor**
☐ Grunnskole	☐ Grunnskole
☐ Videregående skole (gymnas/yrkesskole)	☐ Videregående skole (gymnas/yrkesskole)
☐ Høgskole/universitet (3 år eller mindre)	☐ Høgskole/universitet (3 år eller mindre)
☐ Høgskole/universitet (3 år eller mer)	☐ Høgskole/universitet (3 år eller mer)
☐ Vet ikke	☐ Vet ikke

4. Hvem bor du sammen med (her kan du sette flere kryss)?

☐ Mor
☐ Far
☐ Stemor
☐ Stefar
☐ Omtrent like mye hos mor og far
☐ På hybel/leilighet
☐ Internat
☐ Annet

5. Hvilke karakterer hadde du ved siste karakteroppgjør i følgende fag?

	**6**	**5**	**4**	**3**	**2**	**1**	**Ikke karakter**
Engelsk	☐	☐	☐	☐	☐	☐	☐
Matematikk	☐	☐	☐	☐	☐	☐	☐
Norsk	☐	☐	☐	☐	☐	☐	☐
Kroppsøving	☐	☐	☐	☐	☐	☐	☐

Spørsmål om fysisk aktivitet

6. Hvordan vil du beskrive ditt eget fysiske aktivitetsnivå? (*Passiv* betyr inaktiv/lite hverdagsbevegelse)

☐ Meget aktiv ☐ Middels ☐ Noe aktiv ☐ Litt passiv ☐ Meget passiv

7. Hvordan vil du beskrive din mors fysiske aktivitetsnivå?

☐ Meget aktiv ☐ Middels ☐ Noe aktiv ☐ Litt passiv ☐ Meget passiv

8. Hvordan vil du beskrive din fars fysiske aktivitetsnivå?

☐ Meget aktiv ☐ Middels ☐ Noe aktiv ☐ Litt passiv ☐ Meget passiv

9. Hvordan kommer du deg vanligvis til/fra skolen?

Går	☐
Sykler	☐
Buss	☐
Bil	☐
Motorsykkel, scooter eller moped	☐
Annet, ____________________	☐

10. Hvor lang tid bruker du vanligvis til skolen?

Mindre enn 5 minutter ☐
6 til 15 minutter ☐
16 til 30 minutter ☐
31 minutter til 1 time ☐
Mer enn 1 time ☐

De neste spørsmålene dreier seg om fysisk aktivitet som du gjør på FRITIDEN (for eksempel i helgene, på ettermiddag/kveld og i ferier), IKKE når du er på skolen. Eksempler på fysisk aktivitet er å løpe, gå fort, gå på rulle skøyter, sykle, sparkesykle, gå på ski, svømme, spille fotball eller danse.
Med IDRETT/MOSJON/FYSISK AKTIVITET mener vi all fysisk aktivitet som gjør deg andpusten eller litt svett.

11. Utenom skoletid: Hvor mange ganger i uka driver du idrett/mosjon slik at du blir andpusten eller svett?

☐ ganger per uke

12. Omtrent hvor mange timer til sammen per uke bruker du på dette?

0 timer ☐
1-2 timer ☐
3-4 timer ☐
5-7 timer ☐
8-10 timer ☐
11 timer eller mer ☐

13. Hvor anstrengende pleier idretts-/mosjonsaktivitetene du driver å være? *(Sett bare ett kryss)*

Driver ikke idrett/mosjon ☐
Litt anstrengende ☐
Ganske anstrengende ☐
Meget anstrengende ☐
Svært anstrengende ☐

14. Hvor ofte har du i gjennomsnitt drevet med følgende treningsaktiviteter i løpet av de siste 12 måneder? *(Sett ett kryss for hver aktivitetsgruppe)*

	Aldri	Under 1 gang pr uke	1 gang pr uke	Flere ganger pr uke
**U**tholdenhetsidrett (*feks løp, sykling, langrenn, svømming*)……	☐	☐	☐	☐
**L**ag-/ballidretter (*feks squash, håndball, fotball, ishockey*)……	☐	☐	☐	☐
**S**tyrkeidrett (*feks bryting, vekttrening*)……	☐	☐	☐	☐
**K**ampsport (*feks judo, karate, taekwondo*)……	☐	☐	☐	☐
**T**ekniske idretter (*feks ridning, alpint, telemark, friidrett, snowboard, golf, rullebrett/skøyter*)……	☐	☐	☐	☐
**R**isikoidrett (*feks elvepadling, fjellklatring, paragliding*)……	☐	☐	☐	☐
**A**nnet, spesifiser____________________	☐	☐	☐	☐

I det neste spørsmålet brukes begrepet REGELMESSIG. Da mener vi 3 ganger eller mer i uka, i minst 20 minutter hver gang.

15. Hvilket av disse passer best for deg? *(Sett ett kryss)*

☐ For tiden er jeg ikke fysisk aktiv, og jeg har ingen planer om å bli det i løpet av de neste 6 måneder
☐ For tiden er jeg ikke fysisk aktiv, men jeg tenker å bli mer fysisk aktiv i løpet av de neste 6 måneder
☐ For tiden er jeg noe fysisk aktiv, men det er ikke regelmessig
☐ For tiden er jeg regelmessig fysisk aktiv, men det er først i de siste 6 måneder at jeg har begynt med det
☐ For tiden er jeg regelmessig fysisk aktiv, og jeg har vært det lengre enn 6 måneder

16. Hvis du er fysisk aktiv, hvorfor driver du med fysisk aktivitet? (*Sett et kryss for hvert svaralternativ som rangeres fra helt uenig til helt enig*).

	**Helt uenig** 1	2	3	4	**Helt enig** 5
For å forebygge livsstilssykdommer/plager	☐	☐	☐	☐	☐
For å redusere/vedlikeholde vekt	☐	☐	☐	☐	☐
For å øke muskelmassen/bli sterk	☐	☐	☐	☐	☐
For å bedre mitt utseende	☐	☐	☐	☐	☐
Fordi det er moro	☐	☐	☐	☐	☐
Fordi det er sosialt	☐	☐	☐	☐	☐
For å være mer motstandsdyktig mot sykdommer/skader	☐	☐	☐	☐	☐
For å forbedre humøret	☐	☐	☐	☐	☐
For å få mer overskudd	☐	☐	☐	☐	☐
Vet ikke helt hvorfor jeg er fysisk aktiv	☐	☐	☐	☐	☐
Jeg får dårlig samvittighet hvis jeg ikke er aktiv	☐	☐	☐	☐	☐
Andre synes at jeg må være fysisk aktiv	☐	☐	☐	☐	☐
Ikke noen bestemt grunn	☐	☐	☐	☐	☐

Annet, vennligst noter: ____

17. a) Driver du noen form for organisert idrett nå (idrettslag, konkurranser)?

☐ Ja ☐ Nei

Hvis Ja, vennligst spesifiser hvilke(n) idrett(er)______________________________

Hvis Nei, gå til spørsmål 18.

b) Hvor mye har du trent (totalt, både organisert og egentrening) i snitt i uka de siste 4 ukene?

☐ Under 3 timer
☐ 3 - 5 timer
☐ 6-10 timer
☐ 11-15 timer
☐ 16-20 timer
☐ 21 timer eller mer

18. Når står du vanligvis opp en skoledag?

Før 6.30 ☐
Mellom 6.30 og 7.00 ☐
Mellom 7.00 og 7.30 ☐
Etter 7.30 ☐

19. Når legger du deg vanligvis kvelden før en skoledag?

Før 20.00 ☐
Mellom 20.00 og 21.00 ☐
Mellom 21.00 og 22.00 ☐
Mellom 22.00 og 23.00 ☐
Mellom 23.00 og 24.00 ☐
Etter midnatt ☐

20. Hvor mange timer ser du på TV eller driver du med PC/dataspill på en *vanlig* ukedag (utenom skoletid)?

☐ Jeg ser ikke på TV/driver med dataspill på en vanlig ukedag
☐ Mindre enn 1 time per dag
☐ 1 time per dag
☐ 2 timer per dag
☐ 3 timer per dag
☐ 4 timer per dag
☐ 5 timer eller mer per dag

21. Hvilke av følgende aktiviteter vil du definere som <u>fysisk aktivitet</u>? (*Her kan du sette flere kryss*)

☐ Jogge
☐ Svømme
☐ Klippe plenen
☐ Gå til skolen
☐ Se på TV
☐ Kjøre moped
☐ Danse
☐ Spille volleyball

22. Hva tror du er Helsedirektoratets *minimumsanbefalinger* når det gjelder <u>daglig</u> fysisk aktivitet for ungdom? (*Sett kun ett kryss*).

☐ 15 minutter
☐ 30 minutter
☐ 45 minutter
☐ 60 minutter
☐ 90 minutter
☐ 120 minutter
☐ Vet ikke

Spørsmål om kosthold

23. Hvor mange hovedmåltider spiser du vanligvis per dag? (mellommåltider er ikke iberegnet)

- 1-2 måltider ☐
- 3-4 måltider ☐
- 5 måltider eller mer ☐
- Uregelmessig ☐

24. Spiser du vanligvis noe mellom disse måltidene (mellommåltider)? Ja ☐ Nei ☐

25. Hvor ofte spiser du følgende måltider en vanlig uke? Sett ett kryss for hvert måltid.

	Aldri	1-2 ganger pr uke	3-4 ganger pr uke	5-6 ganger pr uke	Hver dag
Frokost					
Formiddagsmat/lunsj					
Middag					
Kveldsmat					
Mellommåltid/restitusjons-måltid					

26. Hvor ofte spiser du frukt og bær?

- Aldri ☐
- Sjeldnere enn 1 gang i uken ☐
- 1 gang i uken ☐
- 2 ganger i uken ☐
- 3 ganger i uken ☐
- 4 ganger i uken ☐
- 5 ganger i uken ☐
- 6 ganger i uken ☐
- Hver dag ☐
- Flere ganger hver dag ☐

27. Hvor ofte spiser du grønnsaker (inkludert salat)?

- Aldri ☐
- Sjeldnere enn 1 gang i uken ☐
- 1 gang i uken ☐
- 2 ganger i uken ☐
- 3 ganger i uken ☐
- 4 ganger i uken ☐
- 5 ganger i uken ☐
- 6 ganger i uken ☐
- Hver dag ☐
- Flere ganger hver dag ☐

28. Hvor ofte spiser du potet?

- Aldri ☐
- Sjeldnere enn 1 gang i uken ☐
- 1 gang i uken ☐
- 2 ganger i uken ☐
- 3 ganger i uken ☐
- 4 ganger i uken ☐
- 5 ganger i uken ☐
- 6 ganger i uken ☐
- Hver dag ☐
- Flere ganger hver dag ☐

29. Hvor ofte spiser du potetgull, peanøtter o.l.?

- Aldri ☐
- Sjeldnere enn 1 gang i uken ☐
- 1 gang i uken ☐
- 2 ganger i uken ☐
- 3 ganger i uken ☐
- 4 ganger i uken ☐
- 5 ganger i uken ☐
- 6 ganger i uken ☐
- Hver dag ☐
- Flere ganger hver dag ☐

30. Hvor ofte spiser du godterier (sjokolade, blandet godt osv.)?

- Aldri ☐
- Sjeldnere enn 1 gang i uken ☐
- 1 gang i uken ☐
- 2 ganger i uken ☐
- 3 ganger i uken ☐
- 4 ganger i uken ☐
- 5 ganger i uken ☐
- 6 ganger i uken ☐
- Hver dag ☐
- Flere ganger hver dag ☐

31. Hvor ofte drikker du juice?

- Aldri ☐
- Sjeldnere enn 1 gang i uken ☐
- 1 gang i uken ☐
- 2 ganger i uken ☐
- 3 ganger i uken ☐
- 4 ganger i uken ☐
- 5 ganger i uken ☐
- 6 ganger i uken ☐
- Hver dag ☐
- Flere ganger hver dag ☐

32. Hvor ofte drikker du saft MED sukker?

- Aldri ☐
- Sjeldnere enn 1 gang i uken ☐
- 1 gang i uken ☐
- 2 ganger i uken ☐
- 3 ganger i uken ☐
- 4 ganger i uken ☐
- 5 ganger i uken ☐
- 6 ganger i uken ☐
- Hver dag ☐
- Flere ganger hver dag ☐

33. Hvor ofte drikker du saft UTEN sukker

- Aldri ☐
- Sjeldnere enn 1 gang i uken ☐
- 1 gang i uken ☐
- 2 ganger i uken ☐
- 3 ganger i uken ☐
- 4 ganger i uken ☐
- 5 ganger i uken ☐
- 6 ganger i uken ☐
- Hver dag ☐
- Flere ganger hver dag ☐

34. Hvor ofte drikker du brus MED sukker?

- Aldri ☐
- Sjeldnere enn 1 gang i uken ☐
- 1 gang i uken ☐
- 2 ganger i uken ☐
- 3 ganger i uken ☐
- 4 ganger i uken ☐
- 5 ganger i uken ☐
- 6 ganger i uken ☐
- Hver dag ☐
- Flere ganger hver dag ☐

35. Hvor ofte drikker du brus UTEN sukker?

- Aldri ☐
- Sjeldnere enn 1 gang i uken ☐
- 1 gang i uken ☐
- 2 ganger i uken ☐
- 3 ganger i uken ☐
- 4 ganger i uken ☐
- 5 ganger i uken ☐
- 6 ganger i uken ☐
- Hver dag ☐
- Flere ganger hver dag ☐

36. Hvor ofte drikker du vann fra springen?

- Aldri ☐
- Sjeldnere enn 1 gang i uken ☐
- 1 gang i uken ☐
- 2 ganger i uken ☐
- 3 ganger i uken ☐
- 4 ganger i uken ☐
- 5 ganger i uken ☐
- 6 ganger i uken ☐
- Hver dag ☐
- Flere ganger hver dag ☐

37. Hvor ofte drikker du rent kjøpevann uten kullsyre og smak?

- Aldri ☐
- Sjeldnere enn 1 gang i uken ☐
- 1 gang i uken ☐
- 2 ganger i uken ☐
- 3 ganger i uken ☐
- 4 ganger i uken ☐
- 5 ganger i uken ☐
- 6 ganger i uken ☐
- Hver dag ☐
- Flere ganger hver dag ☐

38. Hvor ofte drikker du kjøpevann med kullsyre og/eller smak?

- Aldri ☐
- Sjeldnere enn 1 gang i uken ☐
- 1 gang i uken ☐
- 2 ganger i uken ☐
- 3 ganger i uken ☐
- 4 ganger i uken ☐
- 5 ganger i uken ☐
- 6 ganger i uken ☐
- Hver dag ☐
- Flere ganger hver dag ☐

39. Hvor mange porsjoner frukt og grønnsaker tror du at du spiser hver dag?

- Ingen ☐
- 1 ☐
- 2 ☐
- 3 ☐
- 4 ☐
- 5 ☐
- Mer enn 5 ☐

40. Hvor mange porsjoner frukt og grønnsaker tror du at en på din alder bør spise hver dag?

- Ingen ☐
- 1 ☐
- 2 ☐
- 3 ☐
- 4 ☐
- 5 ☐
- Mer enn 5 ☐

41. Bruker du vanligvis margarin/smør på brødskiven? Ja ☐ Nei ☐

42. Kryss av for om du mener disse påstandene er riktige eller gale (*HUSK kun ett kryss for hvert spørsmål*)

	Riktig	Galt	Vet ikke
Karbohydrater er den viktigste kilden til energi for kroppen	☐	☐	☐
Fett er et næringsstoff som kroppen egentlig ikke trenger	☐	☐	☐
"Fem om dagen" betyr at jeg bør spise 5 måltider om dagen	☐	☐	☐
Fem agurkskiver på brødet teller som en porsjon frukt og grønnsaker	☐	☐	☐
Et jevnt matinntak gir bedre konsentrasjonsevne og økt forbrenning	☐	☐	☐
Blodsukkeret påvirkes både av hva jeg spiser og hvor ofte jeg spiser	☐	☐	☐
Antioksidanter er stoffer som skader kroppen	☐	☐	☐
Kroppen min trenger mat så ofte som hver 3. – 4. time	☐	☐	☐

De neste 3 spørsmålene dreier seg om røyking, snusing og alkohol. Det er viktig at du er ærlig når du svarer på spørsmålet. Husk: Ingen vil se besvarelsen din, verken på skolen eller hjemme.

43. Røyker du?

- Har aldri røykt ☐
- Har prøvd, men røyker ikke i det hele tatt nå ☐
- Har røykt fast, men har sluttet helt nå ☐
- Røyker, men ikke daglig ☐
- Røyker daglig, omtrent _____sigaretter per dag ☐

44. Snuser du?

- Har aldri snust ☐
- Har prøvd, men snuser ikke i det hele tatt nå ☐
- Har snust fast, men har sluttet helt nå ☐
- Snuser, men ikke daglig ☐
- Snuser daglig, omtrent ☐
  _____ poser/priser per dag

45. Har du noen gang drukket øl, vin eller brennevin?

Ja ☐ Nei ☐

Hvis ja, gjennomsnittlig hvor ofte? (*Kryss av for det svaret som passer best til ditt forbruk*).

- Jeg har så vidt smakt alkohol ☐
- Drikker ca. 1 gang per måned ☐
- Drikker ca. 1 gang per uke ☐
- Drikker mer enn en gang per uke ☐

Spørsmål om vekt og helse

Spørsmål 46 og 47 skal kun besvares av gutter, mens spørsmål 48 og 49 kun skal besvares av jenter.

GUTTER:

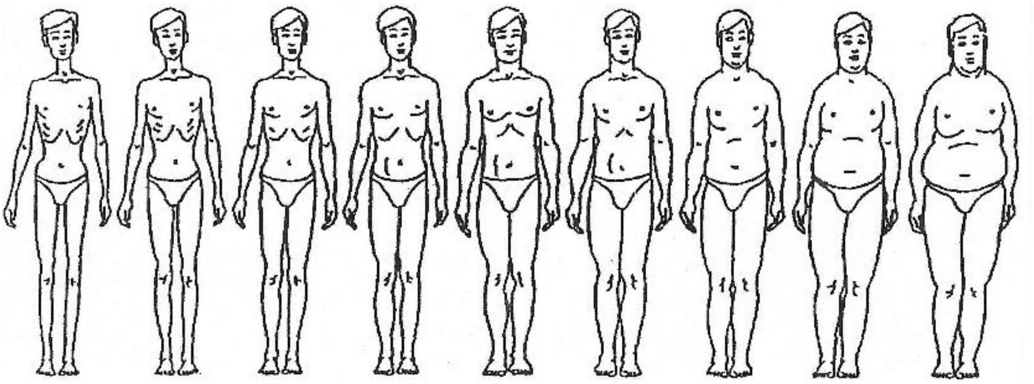

1. 2. 3. 4. 5. 6. 7. 8. 9.

46. Hvilket av bildene over illustrerer best din nåværende kroppsfigur?
Bilde nummer: ☐1 ☐2 ☐3 ☐4 ☐5 ☐6 ☐7 ☐8 ☐9

47. Hvilket av bildene over illustrerer best den kroppsfiguren som du ønsker deg?
Bilde nummer: ☐1 ☐2 ☐3 ☐4 ☐5 ☐6 ☐7 ☐8 ☐9

JENTER:

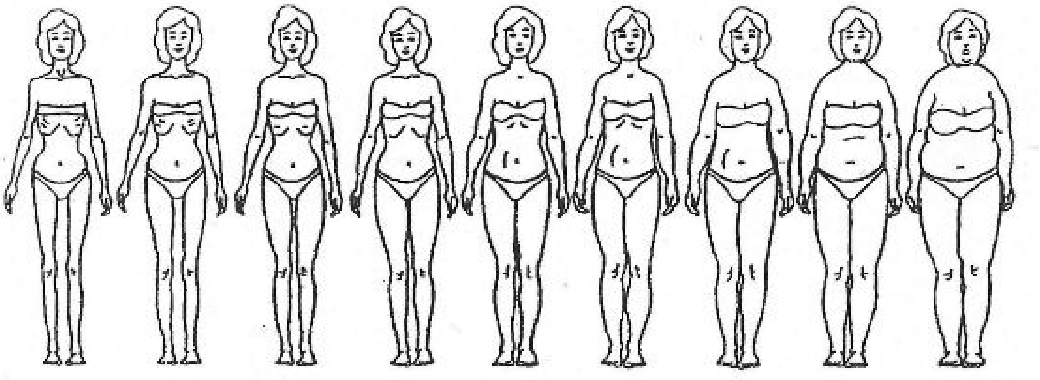

1. 2. 3. 4. 5. 6. 7. 8. 9.

48. Hvilket av bildene over illustrerer best din nåværende kroppsfigur?
Bilde nummer: ☐1 ☐2 ☐3 ☐4 ☐5 ☐6 ☐7 ☐8 ☐9

49. Hvilket av bildene over illustrerer best den kroppsfiguren som du ønsker deg?
Bilde nummer: ☐1 ☐2 ☐3 ☐4 ☐5 ☐6 ☐7 ☐8 ☐9

50.

a) Hvor høy er du? ☐☐☐ Cm

b) Hva er din nåværende vekt ☐☐☐ Kg

51. Hva mener du at din vekt bør være for;

a) å ha det godt med deg selv? ☐☐☐ Kg

b) å prestere bra i din idrett? (dersom du driver organisert idrett) ☐☐☐ Kg

52. Har du noen gang forsøkt å gå opp i vekt? Ja ☐ Nei ☐

53. Har du noen gang forsøkt å sikre at du ikke har gått ned i vekt? Ja ☐ Nei ☐

54. Prøver du å gå opp i vekt nå? Ja ☐ Nei ☐

<u>Hvis nei på spørsmål 52, 53 og 54, gå videre til spørsmål 57.</u>

55. Her følger noen påstander om hvorfor du ønsker/ønsket å gå opp i vekt. Stemmer disse for deg? Sett kryss.

	Ja	Nei
For tynn		
For lett/svak		
For lite muskelmasse (”muskler”)		
Prestere bedre i min idrett		
Se bedre ut		
Helsesøster mener/mente at jeg bør/burde gå opp i vekt		
Legen mener/mente at jeg bør/burde gå opp i vekt		
Trener mener/mente jeg bør/burde gå opp i vekt		
Lærer mener/mente jeg bør/burde gå opp i vekt		
Familiemedlem mener/mente at jeg bør/burde gå opp i vekt		
Venner/kjæreste mener/mente at jeg burde gå opp i vekt		
Lagkamerater/andre utøvere mener/mente jeg bør/burde gå opp i vekt		
Annet: ____________________ ____________________		

56. **Hvilke av følgende metoder har du eventuelt brukt/bruker du for å gå opp i vekt?**

	Alltid	Vanligvis	Ofte	Iblant	Sjelden	Aldri
Økt kalori/energi inntak						
Økt treningsmengde						
Økt treningsintensitet						
Økt treningshyppighet						
Mer styrketrening						
Bruk av kosttilskudd						
Selvkomponert diett						
Diett fra ernæringsfysiolog						
Diett fra andre;______________						
Annen metode: vennligst utdyp: ______________________________ ______________________________						

57. Har du noen gang forsøkt å gå ned i vekt? ☐ Ja ☐ Nei

58. Har du noen gang forsøkt å sikre at du ikke har gått opp i vekt? ☐ Ja ☐ Nei

59. Forsøker du å gå ned i vekt nå? ☐ Ja ☐ Nei

<u>Hvis Nei på spørsmål 57, 58 og 59, gå til spørsmål 64</u>

60. Hvor gammel var du første gang du forsøkte å gå ned i vekt? ☐☐ **År**

61. Hvor mange ganger har du forsøkt å gå ned i vekt?
☐ 1-2 ganger ☐ 3-5 ganger ☐ Mer enn 5 ganger

62. Her følger noen påstander om hvorfor du ønsker/ønsket å gå ned i vekt. Stemmer disse for deg? Sett kryss.

	Ja	Nei
For tung		
For tykk		
Prestere bedre i min idrett (Dersom du driver organisert idrett)		
Se bedre ut		
Helsesøster mener/mente at jeg bør/burde gå ned i vekt		
Legen mener/mente at jeg bør/burde gå ned i vekt		
Lærer mener/mente jeg bør/burde gå ned i vekt		
Trener mener/mente jeg bør/burde gå ned i vekt		
Familiemedlem mener/mente at jeg bør/burde gå ned i vekt		
Venner/kjæreste mener/mente at jeg bør/burde gå ned i vekt		
Lagkamerater/andre utøvere mener/mente jeg bør/burde gå ned i vekt		
Annet:____ ____		

63. Hvilke av følgende metoder har du eventuelt brukt/bruker du for å gå ned i vekt?

	Alltid	Vanligvis	Ofte	Iblant	Sjelden	Aldri
Økt treningsintensitet						
Økt treningsmengde						
Økt treningshyppighet						
Redusert kalori/energi inntak						
Avføringsmidler						
Oppkast						
Slankepiller/pulver						
Vanndrivende midler						
Badstue						
Selvkomponert diett						
Diett fra ernæringsfysiolog						
Diett fra andre;____________ ___________________________						
Annen metode, vennligst utdyp:____________________ ___________________________						

64. Hvor mange år var du da du fikk din første menstruasjon? (Dette spørsmålet besvares kun av jentene).

☐☐ År ☐ Har ikke fått min første menstruasjon ennå

65.
a) Mener du selv du <u>har hatt</u> et spiseproblem? ☐ Ja ☐ Nei ☐ Vet ikke

Hvis ja,

b) Fikk du behandling/oppfølging? ☐ Ja ☐ Nei

66.
a) Mener du selv at du <u>har</u> et spiseproblem? ☐ Ja ☐ Nei ☐ Vet ikke

Hvis ja,
b)Tror du noen vet at du har det? ☐ Ja ☐ Nei ☐ Vet ikke

Hvis ja,
c) Hvem tror du vet det? (*Her kan du sette flere kryss)*.

- ☐ Venner
- ☐ Utøvere i idrettsmiljøet du deltar i (hvis du er utøver selv)
- ☐ Lærer
- ☐ Helsesøster
- ☐ Trener
- ☐ Familie
- ☐ Andre____

67. Har du noen gang blitt fortalt av lege eller psykolog at du har en spiseforstyrrelse?
☐ Ja ☐ Nei

68. Ønsker du å motta tilbud om veiledning i forhold til spiseproblemer?

☐ Ja ☐ Nei ☐ Vet ikke

69. Kjenner du til andre elever på skolen som har eller du antar har en spiseforstyrrelse?

☐ Ja ☐ Nei ☐ Vet ikke

70. Hvilke av følgende påstander mener du er riktige eller gale? (*Sett kun ett kryss for hver påstand*).

	RIKTIG	GALT	VET IKKE
Spiseforstyrrelser er en sykdom som kun handler om mat og spising	☐	☐	☐
Spiseforstyrrelser er en jente/kvinnelidelse	☐	☐	☐
Spiseforstyrrelser er en psykisk lidelse	☐	☐	☐
De som har spiseforstyrrelser er veldig tynne	☐	☐	☐
Man kan bli helt frisk av en spiseforstyrrelse	☐	☐	☐
Idrettsutøvere har redusert risiko for å få spiseforstyrrelser	☐	☐	☐
Jenter har mer kroppsfett enn gutter	☐	☐	☐
Selvtillitt er det samme som "tillitt til egen dyktighet"	☐	☐	☐

Nå kommer siste del av spørreskjemaet – hold konsentrasjonen oppe ☺ ☺

I punktene videre spørres det om dine holdninger, følelser og atferd. Noen av punktene handler om mat og spising. Andre punkter dreier seg om dine følelser i forhold til deg selv. Du skal ved hvert punkt bestemme deg for i hvilken grad utsagnet passer på deg: ALLTID, VANLIGVIS, OFTE, IBLANT, SJELDEN eller ALDRI. Hvis du for eksempel mener at ditt svar på et utsagn er "ofte", sett da et kryss i ruten i kolonnen under "ofte" for det gjeldende utsagn.
Svar på alle punktene og vær sikker på at du setter kryss i den ruten som best beskriver hvordan du føler det **nå for tiden**.
Det er ingen riktige eller gale svar, vær derfor vennlig og svar så ærlig som mulig.

71. For hver påstand, vennligst sett kryss for det alternativet som passer

	Alltid	Vanligvis	Ofte	Iblant	Sjelden	Aldri
1. Jeg spiser søtsaker og karbohydrater uten å bli engstelig						
2. Jeg mener magen min er for stor						
3. Jeg spiser når jeg blir opprørt						
4. Jeg stapper i meg mat						
5. Jeg tenker på slanking						
6. Jeg synes lårene mine er for tykke						
7. Jeg får dårlig samvittighet når jeg har spist for mye						
8. Jeg mener at magen min er passe stor						
9. Jeg er livredd for å legge på meg						
10. Jeg er fornøyd med figuren min						
11. Jeg overvurderer vektens betydning (veldig opptatt av selve kroppsvekten min)						

	Alltid	Vanligvis	Ofte	Iblant	Sjelden	Aldri
12. Jeg har hatt spiseorgier hvor jeg har følt at jeg ikke har kunnet slutte						
13. Jeg er godt fornøyd med overkroppens proporsjoner (hvordan den ser ut)						
14. Jeg liker fasongen på rumpen min						
15. Jeg er svært opptatt av å bli tynnere						
16. Jeg tenker på det å overspise						
17. Jeg mener at hoftene mine er for brede						
18. Sammen med andre spiser jeg moderat, og stapper i meg når jeg er alene						
19. Hvis jeg går opp noen hundre gram i vekt er jeg redd for at jeg vil fortsette å gå opp						
20. Jeg kan få det for meg at jeg skal kaste opp for å gå ned i vekt						
21. Jeg føler at lårene mine er passe store						
22. Jeg mener overkroppen min er for spinkel						
23. Jeg mener at rumpen min er for stor						
24. Jeg spiser eller drikker i hemmelighet						
25. Jeg mener at hoftene mine er akkurat passe brede						
26. Jeg skulle ønske jeg var mer muskuløs						
27. Jeg løfter vekter for å bygge muskler						
28. Jeg bruker proteintilskudd eller andre kosttilskudd						
29. Jeg drikker proteinshaker eller andre energishaker						
30. Jeg prøver å spise så mye jeg kan hver dag						
31. Jeg får dårlig samvittighet hvis jeg mister en treningsøkt						
32. Jeg ville følt meg mer selvsikker hvis jeg hadde større muskler						
33. Andre mennesker synes jeg trener for ofte med vekter						
34. Jeg ville sett bedre ut hvis jeg la på meg mer muskelmasse						
35. Styrketreningsøktene mine gjør at jeg må droppe andre ting i livet mitt						
36. Jeg synes at armene mine ikke er muskuløse nok						
37. Jeg synes at brystkassen min ikke er muskuløs nok						
38. Jeg synes at beina mine ikke er muskuløse nok						

72. Hva synes du egentlig om deg selv? Kryss av for det som passer best for deg (*ett kryss for hvert utsagn*).

	Stemmer svært dårlig	Stemmer nokså dårlig	Stemmer nokså godt	Stemmer svært godt
a) Jeg synes jeg er like smart som andre på min alder				
b) Jeg synes det er ganske vanskelig å få venner				
c) Jeg gjør det bra i all slags sport				
d) Jeg er ofte skuffet over meg selv				
e) Jeg er ganske sein med å bli ferdig med skolearbeidet				
f) Jeg har mange venner				
g) Jeg tror jeg kan gjøre det bra i nesten hvilken som helst ny sport				
h) Jeg liker ikke den måten jeg lever livet mitt på				
i) Jeg gjør det svært godt på skolen				
j) Andre ungdommer har vanskelig for å like meg				
k) Jeg synes jeg er bedre i sport enn andre på min alder				
l) Jeg er stort sett fornøyd med hvordan jeg oppfører meg				
m) Jeg er stort sett fornøyd med meg selv				
n) Jeg har vansker med å svare riktig på skolen				
o) Jeg er populær blant jevnaldrende				
p) Jeg gjør det ikke så godt i nye øvelser i kroppsøvingstimen				
q) Jeg liker meg selv slik jeg er				
r) Jeg betrakter meg selv som ganske intelligent				
s) Jeg føler at jevnaldrende godtar meg				
t) Jeg er svært fornøyd med hvordan jeg er				

TUSEN TAKK FOR AT DU TOK DEG TID TIL UTFYLLING AV SPØRRESKJEMAET!

☺ ☺ ☺
